# The V-shaped association between the ratio of neutrophil counts to prognostic nutritional index and 30-, 60-, and 90-day mortality in elderly critically ill patients aged 65 and older with sepsis: a retrospective study based on the MIMIC database

**DOI:** 10.3389/fnut.2025.1602016

**Published:** 2025-09-03

**Authors:** Jierui Cai, Yifeng Jin, Jiaqi Lou, Bingqing Xu, Huan Qi, Jie Li

**Affiliations:** ^1^Department of General Practice, The First Affiliated Hospital of Soochow University, Suzhou, Jiangsu, China; ^2^Burn Department, Ningbo No. 2 Hospital, Ningbo, Zhejiang, China; ^3^Department of Ultrasound, Children's Hospital of Soochow University, Suzhou, Jiangsu, China

**Keywords:** neutrophil counts, prognostic nutritional index, severe sepsis, elderly patients, mortality risk, V-shaped association

## Abstract

**Objective:**

To investigate the association between the ratio of neutrophil counts to prognostic nutritional index (NPNR) and mortality risk in elderly patients with severe sepsis aged 65 and older.

**Methods:**

This retrospective study utilized data from the MIMIC-IV (v2.2) database. We selected elderly patients (aged ≥65 years) with sepsis admitted to the ICU for the first time. After excluding patients with hematologic disorders, autoimmune diseases, or active malignancies, 13,176 patients were screened. Among them, 1,179 had complete data for NPNR calculation and were included. The primary endpoint was 30-day mortality, with secondary endpoints including 60-day and 90-day in-hospital and ICU mortality. We used Kaplan–Meier survival analysis, Cox proportional hazards models, restricted cubic spline (RCS) regression models, and comprehensive sensitivity analyses to assess the association between NPNR and mortality risk.

**Results:**

NPNR was significantly associated with increased mortality risk. Kaplan–Meier survival curves demonstrated lower survival rates in extreme quartiles (Q1 and Q4) across all time frames (*p* < 0.001). Cox regression showed significant associations for NPNR as a continuous variable and in higher quartiles. After adjustment for confounders including albumin infusion status, the V-shaped association persisted in RCS models (*p* < 0.001). Sensitivity analyses using multiple imputation and subgroup stratification confirmed robustness. The excluded patients (*n* = 11,997) showed comparable baseline severity to the included cohort.

**Conclusion:**

NPNR is a significant predictor of mortality in elderly sepsis patients. Its V-shaped association reflects dual risks of immunonutritional depletion and hyperinflammation, highlighting the clinical utility of this composite marker.

## 1 Background

Sepsis, a life-threatening organ dysfunction caused by a dysregulated host response to infection, remains a significant cause of morbidity and mortality worldwide, particularly in the elderly population. The pathophysiology of sepsis involves a complex interplay of pro-inflammatory and anti-inflammatory responses (1), leading to tissue damage, organ dysfunction, and ultimately death if not treated promptly. With the global aging trend, the incidence of sepsis in older patients has been rising steadily, posing substantial challenges to healthcare systems (1, 2). Early identification of patients at high risk of adverse outcomes is crucial for timely intervention and improved survival rates.

The neutrophil count plays a pivotal role in the body’s defense mechanism against infections (3). Both neutrophilia and neutropenia are recognized indicators of sepsis severity, reflecting hyperinflammatory states and bone marrow exhaustion, respectively, (4, 5). In clinical practice, an elevated neutrophil count is often used as a diagnostic criterion for sepsis and is associated with increased mortality rates (5). However, relying solely on neutrophil counts may not provide a complete picture of a patient’s prognosis, as it does not account for other critical factors such as nutritional status and overall immune function.

The Prognostic Nutritional Index (PNI) was developed to assess the nutritional and immune status of patients, which are closely related to clinical outcomes (6). Serum albumin, a component of PNI, is a marker of nutritional status and is often reduced in patients with chronic diseases or acute infections (7). Lymphocytes, the other component of PNI, are essential for adaptive immune responses, and their depletion can indicate immunosuppression (8). A low PNI has been shown to be an independent risk factor for mortality in various clinical settings, including cancer, surgery, and chronic diseases.

Combining these two markers into a single ratio offers a more holistic view of a patient’s condition. The neutrophil counts/PNI ratio not only reflects the intensity of the inflammatory response but also incorporates the patient’s nutritional and immune status, providing a more nuanced assessment of their risk for adverse outcomes. This integrated approach may overcome limitations of single-parameter models by simultaneously capturing dysregulated inflammation (via neutrophils) and host resilience capacity (via albumin and lymphocytes). This integrated approach may be particularly useful in elderly patients with severe sepsis, who often have complex comorbidities and varying degrees of immune dysfunction (9). Given the growing elderly population and the associated rise in sepsis cases, there is an urgent need to explore novel prognostic markers that can accurately stratify patients based on their risk profiles (10).

Despite emerging evidence on NPNR in oncology and surgery, its prognostic value in elderly sepsis remains underexplored. Crucially, no previous study has comprehensively examined the non-linear relationship between NPNR and mortality in this population, particularly addressing the U-shaped or V-shaped associations suggested by pathophysiological principles. Combining these two markers into a single ratio offers a more holistic view of a patient’s condition. The neutrophil counts/PNI ratio not only reflects the intensity of the inflammatory response but also incorporates the patient’s nutritional and immune status, providing a more nuanced assessment of their risk for adverse outcomes. While previous study has explored ratios of inflammatory and nutritional markers (e.g., neutrophil-to-lymphocyte ratio) in sepsis (11), the neutrophil/PNI ratio specifically has rarely been evaluated. Xie et al. reported that combining neutrophil-related indices with PNI improved prognostic accuracy in sepsis-induced kidney injury, but its role in elderly septic patients remains unclear (11). This integrated approach may be particularly useful in elderly patients with severe sepsis, who often have complex comorbidities and varying degrees of immune dysfunction (9). Given the growing elderly population and the associated rise in sepsis cases, there is an urgent need to explore novel prognostic markers that can accurately stratify patients based on their risk profiles (10).

## 2 Methods

### 2.1 Data source

The dataset utilized in this investigation was sourced from the Medical Information Mart for Intensive Care IV edition 2.2 [MIMIC-IV (v2.2)] repository, a collaborative project by Beth Israel Deaconess Medical Center (BIDMC) and Massachusetts Institute of Technology (MIT) (1, 2). This repository encompasses comprehensive records from BIDMC for all patients who were admitted to either the emergency department or an ICU between 2008 and 2019, with all patient Death information was followed up for 1 year after discharge from the hospital. To protect patient privacy, all personal information was de-identified using randomized codes instead of patient identification; therefore, we did not require patients’ informed consent and ethical approval. The MIMIC-IV (v2.2) database can be downloaded from the Physionet Online Forum [MIMIC-IV v2.2 (physionet.org)] (1). To apply the database to clinical research, the one of co-authors of this study, Jiaqi Lou, completed the Collaborative Institutional Training Initiative (CITI) course and passed the “Conflict of Interest” and “Data or Sample Study” exams (ID: 60691748). After signing the data use agreement, the research team was authorized to use the database and extract the data.

### 2.2 Study population

The MIMIC-IV (v2.2) database contains records of 431,231 hospitalizations and 73,181 ICU admissions. For this study, we selected elderly patients (aged ≥65 years) with sepsis who were admitted to the ICU for the first time (*n* = 13,176). Patients with conditions affecting neutrophil dynamics were excluded: hematologic disorders (ICD-10: D50-D89), autoimmune diseases (ICD-10: M30-M36), and active malignancies (ICD-10: C00-C97) (*n* = 287 excluded). Among the remaining, we identified patients with available data on neutrophil counts, serum albumin levels, and lymphocyte counts measured within 24 h of ICU admission to capture early sepsis phase biology (*n* = 1,180). Patients who stayed in the hospital for less than 24 h were excluded from the study. For the remaining participants, we collected demographic information from their first admission, as well as in-hospital vital signs, laboratory test results, and data on comorbid conditions. The diagnosis of sepsis was based on the presence of a presumed or confirmed infection, along with a Sequential Organ Failure Assessment (SOFA) score of 2 or higher (1, 2). The identification of patients was done using the International Classification of Diseases, 9th and 10th edition (ICD-9 and ICD-10) codes (12).

To address concerns about selection bias, we compared baseline characteristics between included patients (*n* = 1,179) and those excluded due to missing data (*n* = 11,997). No significant differences were found in age (77.1 ± 8.1 vs. 76.8 ± 8.3 years, *p* = 0.15), SOFA score (5.1 ± 2.6 vs. 5.3 ± 2.8, *p* = 0.12), or prevalence of major comorbidities (all *p* > 0.05), supporting the representativeness of our cohort.

### 2.3 Data extraction and variable selection

All data are extracted from MIMIC-IV by executing structured query language (SQL), and the data is extracted from MIMIC-IV by using Navicat Premium structured query language (1, 2), including the baseline demographic variables, vital signs, comorbidities, disease scores, therapeutic interventions, and laboratory tests. Baseline demographic variables consist of age, weight, height, gender, insurance type, language, marital status, and race. Comorbidities include diabetes, heart failure, myocardial infarction, tumors, chronic kidney disease, acute renal failure, liver cirrhosis, hepatitis, tuberculosis, pneumonia, stroke, hyperlipidemia, and chronic obstructive pulmonary disease. Therapeutic interventions involve continuous renal replacement therapy and mechanical ventilation. Administration of albumin preparations within 48 h prior to laboratory measurement was specifically extracted, with 215 patients (18.2%) receiving such treatment. Laboratory tests cover white blood cell count, red blood cell count, neutrophil count, lymphocyte count, platelet count, hemoglobin level, red cell distribution width, hematocrit, albumin, globulin, total protein, chloride, glucose, glycated hemoglobin, triglycerides, total cholesterol, high-density lipoprotein, low-density lipoprotein, alanine aminotransferase, and aspartate aminotransferase. All laboratory parameters were obtained from the first measurement within 24 h of ICU admission. In the laboratory test results, the Neutrophil counts/Prognostic Nutritional Index was determined using the formula (6): Neutrophil counts/Prognostic Nutritional Index = Neutrophil counts (×10^9^/L)/[serum albumin (g/dL) + 5 × lymphocyte counts (×10^9^/L)].

### 2.4 Outcomes and measures

The primary endpoint of this study was 30-day mortality, defined as the time from ICU admission to death. Secondary endpoints covered in-hospital and in-ICU mortality at 60- and 90-day. Also, the durations of hospital and ICU stays, both measured in days, were recorded.

### 2.5 Statistical analysis

Continuous variables were tested for normality using the Shapiro–Wilk test. Continuous variables are presented as mean ± standard deviation (SD) for normally distributed data and as median [interquartile range (IQR)] for non-normally distributed data. Categorical variables are presented as counts and percentages (%). To compare group differences, categorical variables were analyzed using Fisher’s exact test or chi-square test, while continuous variables were evaluated using Student’s t-test (for normally distributed data) or the Mann–Whitney U test (for non-normally distributed data) (12).

Kaplan–Meier survival analysis was used to evaluate the incidence of endpoint events between different ratio of neutrophil counts to prognostic nutritional index levels, and the differences were assessed using log rank test (1).

The Cox proportional hazards model was used to calculate the hazard ratio (HR) and 95% confidence interval (CI) between the ratio of neutrophil counts to prognostic nutritional index and the endpoint (13). The ratio of neutrophil counts to prognostic nutritional index was used as a continuous variable and quartiles, respectively, and models were adjusted for confounding factors. We incorporated clinical and prognostic variables into multivariate models: Model 1: Univariate analysis; Model 2: Based on Model 1, adjusted for age, gender, height, weight, race, insurance, language, and marital status; Model 3: Further adjustments on the basis of Model 2 to include albumin infusion, AKI, continuous renal replacement therapy, mechanical ventilation, hypertension, type 2 diabetes, heart failure, chronic kidney disease, acute renal failure, hepatitis, hyperlipidemia, stroke, WBC, RBC, hemoglobin, RDW, globulin, chloride, glucose, and albumin infusion.

In addition, we used restricted cubic spline (RCS) regression models to analyze the non-linear correlation between baseline ratio of neutrophil counts to prognostic nutritional index and mortality in hospital and ICU. The ratio of neutrophil counts to prognostic nutritional index was input as a continuous variable into the model (with the median value as the reference). We tested models with 3–5 knots to assess curve shape stability. We calculated the *p*-value of the trend using the quartile level (14). Unadjusted RCS results are provided in Supplementary Figure 1.

Perform subgroup analysis to explore whether there are differences between different subgroups based on age (more than or equal to 65 years old and less than 70 years old, more than or equal to 70 years old and less than 80 years old, more than or equal to 80 years old and less than 90 years old, more than or equal to 90 years old), sex, BMI (<27.4 kg/m^2^, 27.4–31.2 kg/m^2^, ≥31.2 kg/m^2^), Hypertension, Type 2 Diabetes, Malignant tumor, Chronic kidney disease, Acute renal failure, Hepatitis, Hyperlipidemia, Continuous renal replacement therapy and Mechanical ventilation, in order to determine the consistency of the prognostic value of the ratio of neutrophil counts to prognostic nutritional index for the primary outcome. Subgroup analysis also used Cox models to adjust for all variables in the patient’s baseline information (15, 16). Perform subgroup analysis using odds ratio (OR). The reason is that when the dependent variable is a binary variable (such as death/survival), OR is a suitable statistical indicator that can well reflect the association between exposure factors and outcome events. OR can complement HR in Cox regression models, which is mainly used to analyze survival time data, while OR can be used to analyze differences in mortality risk among different subgroups at specific time points, providing a comprehensive risk assessment together. And in subgroup analysis, OR facilitates the comparison of effect differences between different subgroups. By observing the OR values and their confidence intervals of each subgroup, it is possible to quickly determine whether the prospective values of NPNR ratios in different subgroups are consistent.

In terms of handling missing data, multiple imputation with the random forest method (R package “mice”) (17) was employed to generate 10 imputed datasets. Before imputation, variables with over 50% missingness were excluded. Supplementary Table S1 presents a comparison of baseline characteristics before and after imputation, indicating no significant differences. Data processing and analysis were conducted using R version 4.3.0, with a statistically significant difference defined as *p* < 0.05 on both sides.

## 3 Results

### 3.1 Characteristics of included patients

**Figure 1:**
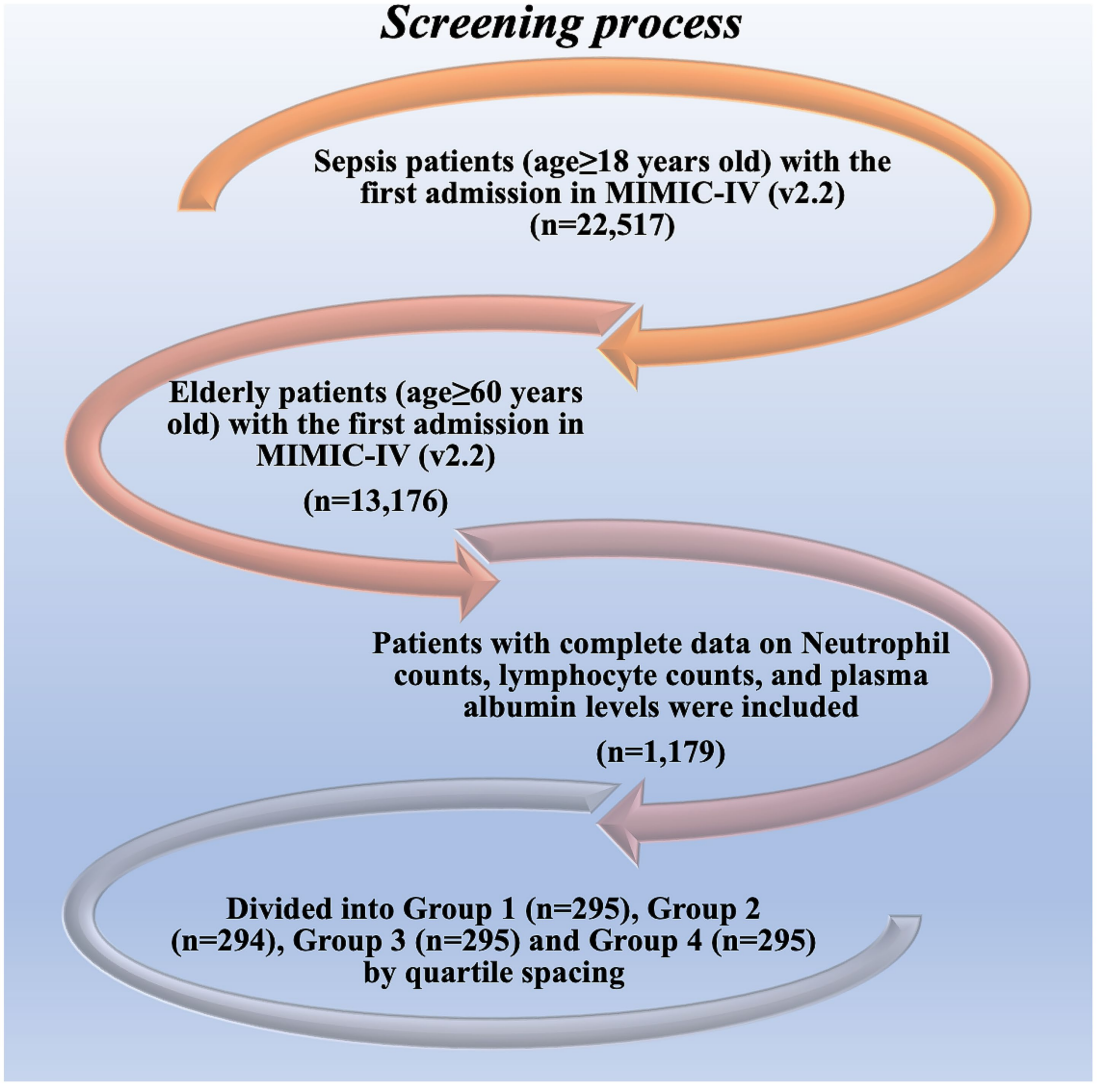
Selection of the study population from the MIMIC-IV database.

Among the adult patients in the MIMIC-IV database, 22,517 subjects met our eligibility criteria. Different forecast factors are extracted from the database. Data cleaning was performed on the included predictors. Among 13,176 elderly sepsis patients aged 65 and above, after excluding 287 patients with hematologic/autoimmune/oncologic disorders, the final number of patients who met the data integrity requirements was 1,179, including 295 people in Group 1 (Q1), accounting for 25.02%, 294 people in Group 2 (Q2), accounting for 24.94%, 295 people in Group 3 (Q3), accounting for 25.02%, and 295 people in Group 4 (Q4), accounting for 25.02%. The screening procedure is shown in Figure 1 and Table 1. Albumin infusion occurred in 215 patients (18.2%), with no significant difference across quartiles (Q1: 19.7%, Q2: 17.0%, Q3: 18.3%, Q4: 18.0%; *p* = 0.32).

**Table 1:** Characteristics and outcomes of participants categorized by the ratio of neutrophil counts to prognostic nutritional index.

Variables	Total (*n* = 1,179)	Group 1 (*n* = 295)	Group 2 (*n* = 294)	Group 3 (*n* = 295)	Group 4 (*n* = 295)	Statistic	*p*
Age, Mean ± SD	77.13 ± 8.10	77.02 ± 8.24	77.44 ± 8.34	77.16 ± 7.96	76.92 ± 7.89	*F* = 0.23	0.872
Weight, Mean ± SD	79.23 ± 22.56	78.23 ± 21.10	77.37 ± 21.47	82.45 ± 24.90	78.84 ± 22.35	*F* = 2.88	**0.035**
Height, Mean ± SD	167.99 ± 10.29	168.71 ± 10.51	166.66 ± 9.88	168.27 ± 10.64	168.33 ± 10.10	*F* = 1.36	0.255
WBC (×10^9^/L)	15.08 ± 12.10	10.86 ± 16.15	11.73 ± 5.36	15.06 ± 5.91	22.67 ± 13.23	*F* = 68.25	**<0.001**
RBC (×10^12^/L)	3.40 ± 0.73	3.36 ± 0.78	3.43 ± 0.72	3.38 ± 0.74	3.42 ± 0.68	*F* = 0.57	0.635
Neutrophil count (×10^9^/L)	12.54 ± 9.83	5.50 ± 3.99	9.24 ± 4.29	13.32 ± 5.06	22.09 ± 13.22	*F* = 255.01	**<0.001**
Lymphocytes (×10^9^/L)	1.34 ± 4.34	2.53 ± 8.50	1.17 ± 0.82	0.96 ± 0.58	0.69 ± 0.49	*F* = 10.67	**<0.001**
Platelet count (×10^9^/L)	186.73 ± 103.12	156.75 ± 98.83	189.13 ± 91.04	190.90 ± 99.35	210.13 ± 115.06	*F* = 14.04	**<0.001**
Hemoglobin (g/dL)	10.05 ± 2.06	9.95 ± 2.03	10.13 ± 2.00	10.09 ± 2.22	10.04 ± 2.00	*F* = 0.40	0.750
RDW (%)	15.95 ± 2.73	15.99 ± 3.11	15.67 ± 2.57	15.78 ± 2.40	16.35 ± 2.75	*F* = 3.58	**0.014**
Hematocrit (%)	31.21 ± 6.27	30.91 ± 6.53	31.37 ± 6.05	31.33 ± 6.56	31.23 ± 5.96	*F* = 0.32	0.811
Albumin (g/L)	3.02 ± 0.63	3.16 ± 0.62	3.19 ± 0.61	3.00 ± 0.60	2.72 ± 0.57	*F* = 37.25	**<0.001**
Globulin (g/dL)	2.85 ± 1.34	3.14 ± 1.86	2.61 ± 0.76	2.76 ± 0.47	2.40 ± 0.83	*F* = 0.90	0.449
Total protein (g/dL)	5.95 ± 1.37	6.20 ± 1.80	5.77 ± 1.09	6.00 ± 0.83	5.33 ± 0.82	*F* = 1.12	0.344
Chlorine (mmol/L)	102.95 ± 6.40	103.72 ± 6.78	103.01 ± 6.27	102.51 ± 6.46	102.58 ± 6.02	*F* = 2.23	0.083
Glucose (mmol/L)	153.12 ± 65.23	147.06 ± 60.10	152.07 ± 59.86	157.85 ± 74.52	155.49 ± 65.14	*F* = 1.52	0.206
A1c (%)	6.35 ± 1.61	6.25 ± 1.33	6.57 ± 1.79	6.26 ± 1.61	6.35 ± 1.93	*F* = 0.44	0.727
Triglyceride (mg/dL)	150.83 ± 119.62	153.40 ± 131.49	134.34 ± 85.52	150.02 ± 119.73	164.29 ± 130.48	F = 0.44	0.725
Total cholesterol Glucose (mmol/L)	145.93 ± 58.54	153.53 ± 59.19	135.72 ± 41.18	152.57 ± 69.68	129.09 ± 61.22	*F* = 1.14	0.336
High-density lipoprotein (mg/dL)	48.06 ± 20.74	47.46 ± 19.76	46.82 ± 18.72	50.36 ± 24.52	47.93 ± 21.45	*F* = 0.16	0.924
Low-density lipoprotein (mg/dL)	78.46 ± 47.43	86.16 ± 48.19	67.29 ± 33.97	82.21 ± 59.71	69.14 ± 38.44	*F* = 1.15	0.333
ALT (U/L)	140.52 ± 430.07	100.75 ± 329.53	106.45 ± 288.42	172.56 ± 602.64	181.91 ± 426.37	*F* = 2.86	**0.036**
AST (U/L)	280.81 ± 1,206.46	184.83 ± 777.44	202.69 ± 720.31	413.67 ± 1,981.03	321.49 ± 866.12	*F* = 2.31	0.074
CRRT (Day)	5.11 ± 4.76	3.85 ± 3.20	5.05 ± 3.06	6.03 ± 5.55	4.89 ± 5.56	*F* = 0.93	0.428
Ventilation (h)	78.84 ± 98.02	74.63 ± 91.87	75.42 ± 93.17	89.88 ± 105.64	75.25 ± 100.37	*F* = 1.40	0.241
Gender, *n* (%)						*χ*^2^ = 4.67	0.198
Female	532 (45.12)	146 (49.49)	136 (46.26)	121 (41.02)	129 (43.73)		
Man	647 (54.88)	149 (50.51)	158 (53.74)	174 (58.98)	166 (56.27)		
Insurance, *n* (%)						*χ*^2^ = 7.86	0.249
Medicaid	24 (2.04)	5 (1.69)	2 (0.68)	6 (2.03)	11 (3.73)		
Medicare	758 (64.29)	193 (65.42)	195 (66.33)	189 (64.07)	181 (61.36)		
Other	397 (33.67)	97 (32.88)	97 (32.99)	100 (33.90)	103 (34.92)		
Language, *n* (%)						*χ*^2^ = 1.86	0.602
Others	129 (10.94)	35 (11.86)	26 (8.84)	33 (11.19)	35 (11.86)		
English	1,050 (89.06)	260 (88.14)	268 (91.16)	262 (88.81)	260 (88.14)		
Marital status, *n* (%)						*χ*^2^ = 13.20	0.355
Divorced	82 (6.96)	26 (8.81)	23 (7.82)	16 (5.42)	17 (5.76)		
Married	498 (42.24)	142 (48.14)	115 (39.12)	127 (43.05)	114 (38.64)		
Other	187 (15.86)	37 (12.54)	49 (16.67)	49 (16.61)	52 (17.63)		
Single	210 (17.81)	44 (14.92)	54 (18.37)	53 (17.97)	59 (20.00)		
Widowed	202 (17.13)	46 (15.59)	53 (18.03)	50 (16.95)	53 (17.97)		
CRRT, *n* (%)						*χ*^2^ = 6.85	0.077
Yes	105 (8.91)	20 (6.78)	21 (7.14)	36 (12.20)	28 (9.49)		
No	1,074 (91.09)	275 (93.22)	273 (92.86)	259 (87.80)	267 (90.51)		
Ventilation, *n* (%)						*χ*^2^ = 0.49	0.921
Yes	977 (82.87)	241 (81.69)	245 (83.33)	247 (83.73)	244 (82.71)		
No	202 (17.13)	54 (18.31)	49 (16.67)	48 (16.27)	51 (17.29)		
Hypertension *n* (%)						*χ*^2^ = 13.08	**0.004**
No	746 (63.27)	162 (54.92)	195 (66.33)	201 (68.14)	188 (63.73)		
Yes	433 (36.73)	133 (45.08)	99 (33.67)	94 (31.86)	107 (36.27)		
Type 2 diabetes mellitus *n* (%)						*χ*^2^ = 9.00	**0.029**
No	778 (65.99)	200 (67.80)	176 (59.86)	192 (65.08)	210 (71.19)		
Yes	401 (34.01)	95 (32.20)	118 (40.14)	103 (34.92)	85 (28.81)		
Heart failure *n* (%)						*χ*^2^ = 13.96	**0.003**
No	698 (59.20)	195 (66.10)	158 (53.74)	160 (54.24)	185 (62.71)		
Yes	481 (40.80)	100 (33.90)	136 (46.26)	135 (45.76)	110 (37.29)		
Myocardial infarction *n* (%)						*χ*^2^ = 7.65	0.054
No	922 (78.20)	244 (82.71)	217 (73.81)	226 (76.61)	235 (79.66)		
Yes	257 (21.80)	51 (17.29)	77 (26.19)	69 (23.39)	60 (20.34)		
Malignant tumor *n* (%)						*χ*^2^ = 6.81	0.078
No	932 (79.05)	235 (79.66)	237 (80.61)	242 (82.03)	218 (73.90)		
Yes	247 (20.95)	60 (20.34)	57 (19.39)	53 (17.97)	77 (26.10)		
Chronic kidney disease *n* (%)						*χ*^2^ = 8.04	**0.045**
No	824 (69.89)	222 (75.25)	190 (64.63)	204 (69.15)	208 (70.51)		
Yes	355 (30.11)	73 (24.75)	104 (35.37)	91 (30.85)	87 (29.49)		
Acute renal failure *n* (%)						*χ*^2^ = 30.86	**<0.001**
No	460 (39.02)	142 (48.14)	134 (45.58)	91 (30.85)	93 (31.53)		
Yes	719 (60.98)	153 (51.86)	160 (54.42)	204 (69.15)	202 (68.47)		
Cirrhosis *n* (%)						*χ*^2^ = 0.96	0.811
No	1,068 (90.59)	265 (89.83)	270 (91.84)	268 (90.85)	265 (89.83)		
Yes	111 (9.41)	30 (10.17)	24 (8.16)	27 (9.15)	30 (10.17)		
Hepatitis *n* (%)						*χ*^2^ = 0.17	0.982
No	1,114 (94.49)	279 (94.58)	279 (94.90)	278 (94.24)	278 (94.24)		
Yes	65 (5.51)	16 (5.42)	15 (5.10)	17 (5.76)	17 (5.76)		
Pneumonia *n* (%)						*χ*^2^ = 10.73	**0.013**
No	698 (59.20)	194 (65.76)	167 (56.80)	157 (53.22)	180 (61.02)		
Yes	481 (40.80)	101 (34.24)	127 (43.20)	138 (46.78)	115 (38.98)		
Stroke *n* (%)						*χ*^2^ = 3.61	0.306
No	1,076 (91.26)	277 (93.90)	267 (90.82)	265 (89.83)	267 (90.51)		
Yes	103 (8.74)	18 (6.10)	27 (9.18)	30 (10.17)	28 (9.49)		
Hyperlipemia *n* (%)						*χ*^2^ = 1.90	0.593
No	652 (55.30)	169 (57.29)	156 (53.06)	158 (53.56)	169 (57.29)		
Yes	527 (44.70)	126 (42.71)	138 (46.94)	137 (46.44)	126 (42.71)		
COPD *n* (%)						*χ*^2^ = 8.40	**0.038**
No	964 (81.76)	257 (87.12)	238 (80.95)	238 (80.68)	231 (78.31)		
Yes	215 (18.24)	38 (12.88)	56 (19.05)	57 (19.32)	64 (21.69)		

*F*: ANOVA, *χ*^2^: Chi-square test, −: Fisher exact, SD, standard deviation; ALT, Alanine aminotransferase; AST, Aspartate aminotransferase; CRRT, Continuous Renal Replacement Therapy; COPD, Chronic Obstructive Pulmonary Disease; WBC, White blood cell count; RBC, Red blood cell count; RDW, Red blood cell distribution width; A1c, Glycated hemoglobin A1c or HbA1c*: Simulated *p*-value. Bold font indicates *p*<0.05, the differences have statistical significance.

### 3.2 Kaplan–Meier survival curve analysis

**Figure 2:**
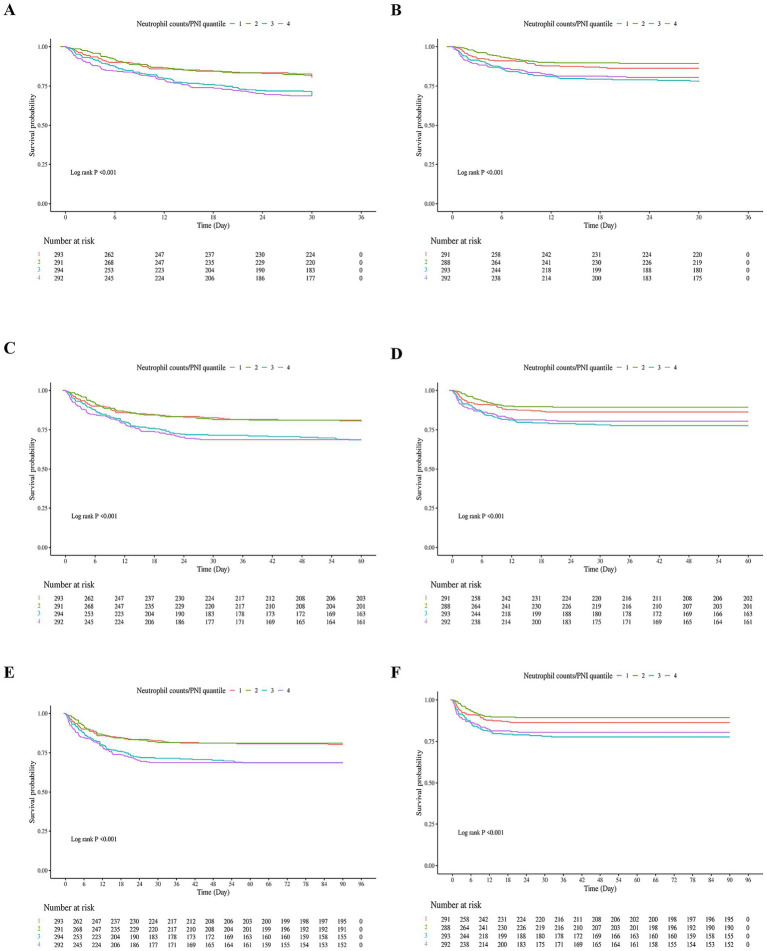
Kaplan–Meier Survival curves for in-hospital and ICU mortality at 30, 60, and 90 days based on neutrophil counts to prognostic nutritional index (neutrophil counts to prognostic nutritional index) quartiles in elderly patients with severe sepsis. **(A)** In-hospital 30-day survival curve. Patients were stratified into four quartiles based on their neutrophil counts to prognostic nutritional index values. The lowest quartile (Q1) represents patients with the lowest neutrophil counts to prognostic nutritional index values, while the highest quartile (Q4) represents those with the highest neutrophil counts to prognostic nutritional index values. The survival curve shows a significant difference in survival rates across quartiles, with the lowest quartile having the poorest survival and the highest quartile having the best survival. **(B)** ICU 30-day survival curve. Similar to **(A)**, patients were divided into four quartiles based on neutrophil counts to prognostic nutritional index. The survival curve demonstrates a significant difference in survival rates within the ICU setting, with lower quartiles associated with higher mortality. **(C)** In-hospital 60-day survival curve. Patients in the lowest neutrophil counts to prognostic nutritional index quartile continued to have the lowest survival rates compared to higher quartiles. The log-rank test confirmed significant differences in survival across quartiles. **(D)** ICU 60-day survival curve. The survival curve for ICU patients at 60 days shows a consistent pattern where lower neutrophil counts to prognostic nutritional index quartiles are linked to higher mortality rates. **(E)** In-hospital 90-day survival curve. The survival differences between quartiles remained significant, with the lowest quartile having the highest mortality rate over the 90-day period. **(F)** ICU 90-day survival curve. The final panel shows the survival curve for ICU patients at 90 days, reinforcing the prognostic value of neutrophil counts to prognostic nutritional index in predicting mortality within the ICU environment. Number at Risk: Tables below each survival curve indicate the number of patients remaining in the analysis at specific time points. Log-rank *p*-values: All panels had highly significant log-rank *p*-values (<0.001), indicating substantial differences in survival across neutrophil counts to prognostic nutritional index quartiles for both in-hospital and ICU settings at all time points.

The Kaplan–Meier survival curves for in-hospital and ICU mortality at 30, 60, and 90 days, based on the neutrophil/PNI ratio divided into four quartiles, are presented in Figure 2. These analyses included elderly patients with severe sepsis aged 65 and older.

Figure 2A shows the in-hospital 30-day survival curve. 30-day survival rates were: Q1: 58.3%, Q2: 72.1%, Q3: 65.8%, Q4: 61.0%. Patients in the lowest quartile (Q1) had the lowest survival rate, while those in Q2 had the highest. Similar trends were observed in Figure 2B (ICU 30-day), Figure 2C (in-hospital 60-day), Figure 2D (ICU 60-day), Figure 2E (in-hospital 90-day), and Figure 2F (ICU 90-day). Log-rank tests for all panels were highly significant (*p* < 0.001). The fourth quartile (Q4) showed lower survival than Q2–Q3 but higher survival than Q1, which may be attributed to a smaller proportion of patients with severe malnutrition (higher albumin levels) compared to Q1, despite elevated neutrophil counts.

Number at risk tables below each survival curve show the number of patients remaining in the analysis at specific time points. Overall, these results demonstrate a pattern where extreme quartiles (Q1 and Q4) are associated with higher mortality rates, supporting a non-linear relationship.

### 3.3 Cox regression models for all-cause mortality (in hospital and in ICU)

Table 2 presents the results of Cox proportional hazards models examining the association between the ratio of neutrophil counts to prognostic nutritional index and mortality risk in elderly patients with severe sepsis. The analysis was conducted at 30, 60, and 90 days for both in-hospital and ICU mortality, with NPNR evaluated as a continuous variable and across quartiles (Q1–Q4).

**Table 2:** The association between the ratio of neutrophil counts to prognostic nutritional index groups and in-hospital and ICU mortality.

Exposure	Model 1		Model 2		Model 3	
	HR (95% CI)	*p*-value	HR (95% CI)	*p*-value	HR (95% CI)	*p*-value
In-hospital mortality at 30-day						
Neutrophil counts/PNI as continuous	1.06 (1.03–1.08)	<0.001	1.06 (1.03–1.08)	<0.001	1.06 (1.03–1.08)	<0.001
Q1	1.00 (Reference)		1.00 (Reference)		1.00 (Reference)	
Q2	0.96 (0.66–1.39)	0.824	0.83 (0.57–1.22)	0.347	0.9 (0.6–1.35)	0.614
Q3	1.69 (1.21–2.36)	0.002	1.49 (1.06–2.11)	0.024	1.4 (0.97–2.02)	0.074
Q4	1.74 (1.25–2.43)	<0.001	1.58 (1.11–2.24)	0.010	1.50 (1.03–2.19)	0.037
ICU mortality at 30-day						
Neutrophil counts/PNI as continuous	1.05 (1.02–1.08)	0.001	1.05 (1.02–1.08)	0.001	1.05 (1.02–1.08)	0.001
Q1	1.00 (Reference)		1.00 (Reference)		1.00 (Reference)	
Q2	0.76 (0.47–1.23)	0.261	0.64 (0.39–1.04)	0.070	0.64 (0.43–0.95)	0.026
Q3	1.69 (1.13–2.52)	0.010	1.43 (0.95–2.17)	0.088	1.50 (1.11–2.03)	0.009
Q4	1.51 (1.01–2.27)	0.050	1.34 (0.87–2.06)	0.187	1.36 (0.99–1.86)	0.055
In-hospital mortality at 60-day						
Neutrophil counts/PNI as continuous	1.06 (1.03–1.08)	<0.001	1.06 (1.03–1.08)	<0.001	1.06 (1.03–1.08)	<0.001
Q1	1.00 (Reference)		1.00 (Reference)		1.00 (Reference)	
Q2	0.96 (0.66–1.39)	0.822	0.83 (0.57–1.22)	0.344	0.90 (0.6–1.35)	0.605
Q3	1.69 (1.21–2.36)	0.002	1.49 (1.06–2.11)	0.024	1.40 (0.97–2.02)	0.075
Q4	1.74 (1.25–2.43)	0.001	1.58 (1.11–2.24)	0.010	1.50 (1.02–2.19)	0.037
ICU mortality at 60-day						
Neutrophil counts/PNI as continuous	1.05 (1.02–1.08)	0.001	1.05 (1.02–1.08)	0.001	1.05 (1.02–1.08)	0.001
Q1	1.00 (Reference)		1.00 (Reference)		1.00 (Reference)	
Q2	0.76 (0.47–1.23)	0.261	0.64 (0.39–1.04)	0.070	0.64 (0.43–0.95)	0.026
Q3	1.69 (1.13–2.52)	0.010	1.43 (0.95–2.17)	0.088	1.50 (1.11–2.03)	0.009
Q4	1.51 (1.01–2.27)	0.050	1.34 (0.87–2.06)	0.187	1.36 (0.99–1.86)	0.055
In-hospital mortality at 90-day						
Neutrophil counts/PNI as continuous	1.06 (1.03–1.08)	<0.001	1.06 (1.03–1.08)	<0.001	1.06 (1.03–1.08)	<0.001
Q1	1.00 (Reference)		1.00 (Reference)		1.00 (Reference)	
Q2	0.96 (0.66–1.39)	0.822	0.83 (0.57–1.22)	0.344	0.90 (0.60–1.35)	0.605
Q3	1.69 (1.21–2.36)	0.002	1.49 (1.06–2.11)	0.003	1.40 (0.97–2.02)	0.075
Q4	1.74 (1.25–2.43)	0.001	1.58 (1.11–2.24)	<0.001	1.50 (1.02–2.19)	0.037
ICU mortality at 90-day						
Neutrophil counts/PNI as continuous	1.05 (1.02–1.08)	0.001	1.05 (1.02–1.08)	0.001	1.05 (1.02–1.08)	0.001
Q1	1.00 (Reference)		1.00 (Reference)		1.00 (Reference)	
Q2	0.76 (0.43–1.23)	0.261	0.64 (0.39–1.04)	0.070	0.64 (0.43–0.95)	0.026
Q3	1.69 (1.13–2.52)	0.010	1.43 (0.95–2.17)	0.088	1.50 (1.11–2.03)	0.009
Q4	1.51 (1.01–2.27)	0.050	1.34 (0.87–2.06)	0.187	1.36 (0.99–1.86)	0.055

*HR hazard ratio, CI confidential interval.

Model 1 = Cox univariate analysis.

Model 2 = Adjusted for age, gender, height, weight, race, insurance, language and marital status.

Model 3 = Adjusted for age, gender, height, weight, race, insurance, language and marital status, albumin infusion, AKI, Continuous renal replacement therapy, Mechanical ventilation, Hypertension, Type 2 Diabetes, Heart failure, Chronic kidney disease, Acute renal failure, Hepatitis, Hyperlipidemia, stroke, WBC, RBC, Hemoglobin, RDW, globulin, chloride, and glucose.

For 30-day in-hospital mortality, the ratio of neutrophil counts to prognostic nutritional index, when analyzed as a continuous variable, showed a significant association with mortality risk (HR 1.06, 95% CI 1.03–1.08, *p* < 0.001). This finding remained consistent across all models. When evaluated across quartiles, the third and fourth quartiles exhibited significant associations. Specifically, in Model 2, the third quartile had a hazard ratio of 1.49 (95% CI 1.06–2.11, *p* = 0.024), and the fourth quartile had a hazard ratio of 1.58 (95% CI 1.11–2.24, *p* = 0.010). In Model 3, the fourth quartile remained significant with a hazard ratio of 1.50 (95% CI 1.02–2.19, *p* = 0.037) (Table 2).

For 30-day ICU mortality, the ratio as a continuous variable was also significantly associated with mortality risk (HR 1.05, 95% CI 1.02–1.08, *p* = 0.001). The second quartile showed a significant inverse association in Model 3 (HR 0.64, 95% CI 0.43–0.95, *p* = 0.026), while the third quartile had a significant positive association (HR 1.50, 95% CI 1.11–2.03, *p* = 0.009) (Table 2).

At 60 days, the ratio as a continuous variable continued to show a significant association with in-hospital mortality (HR 1.06, 95% CI 1.03–1.08, *p* < 0.001). The third and fourth quartiles maintained significant associations in Model 2, with the fourth quartile remaining significant in Model 3. For ICU mortality, the significant association for the ratio as a continuous variable persisted (HR 1.05, 95% CI 1.02–1.08, *p* = 0.001). The second and third quartiles exhibited similar significant associations as observed in the 30-day ICU mortality analysis (Table 2).

For 90-day in-hospital mortality, the ratio as a continuous variable maintained its significant association (HR 1.06, 95% CI 1.03–1.08, *p* < 0.001). The third and fourth quartiles showed significant associations in Model 2, with the fourth quartile remaining significant in Model 3. In the case of ICU mortality, the ratio as a continuous variable continued to be significantly associated with mortality risk (HR 1.05, 95% CI 1.02–1.08, *p* = 0.001). The second and third quartiles preserved their significant associations from previous time points (Table 2).

The ratio of neutrophil counts to prognostic nutritional index is significantly associated with increased mortality risk in elderly patients with severe sepsis, both as a continuous variable and across higher quartiles. This association persists across different time frames and settings, indicating its robust prognostic value. The subgroup analysis revealed consistent trends across various patient characteristics, further supporting the clinical relevance of the ratio of neutrophil counts to prognostic nutritional index in predicting mortality outcomes.

### 3.4 RCS models for all-cause mortality

We applyed the restricted cubic spline (RCS) regression models to reveal the risk. The RCS regression models in Supplementary Figure 1A show the relationship between the ratio of neutrophil counts to prognostic nutritional index and mortality risk in elderly patients with severe sepsis aged 65 and older. Before adjusting for covariates, Supplementary Figure 1A shows the in-hospital 30-day mortality risk. The curve is relatively flat, with no significant linear (*p* = 0.431) or nonlinear (*p* = 0.905) association. This suggests that neutrophil counts to prognostic nutritional index is not strongly linked to mortality risk at this time point when covariates are not considered. Supplementary Figure 1B presents the ICU 30-day mortality risk. The curve shows minimal fluctuation, indicating no significant nonlinear association (*p* = 0.494). The overall *p*-value (*p* = 0.387) also suggests no significant linear association between neutrophil counts to prognostic nutritional index and mortality risk.

Panel C shows the in-hospital 60-day mortality risk. The curve is relatively flat, with no significant linear (*p* = 0.739) or nonlinear (*p* = 0.967) association. Thus, neutrophil counts to prognostic nutritional index does not seem to influence mortality risk at this time point before covariate adjustment.

Supplementary Figure 1D presents the ICU 60-day mortality risk. The curve shows minimal fluctuation, indicating no significant nonlinear association (*p* = 0.984). The overall *p*-value (*p* = 0.403) also suggests no significant linear association between neutrophil counts to prognostic nutritional index and mortality risk. Supplementary Figure 1E shows the in-hospital 90-day mortality risk. The curve is relatively flat, with no significant linear (*p* = 0.783) or nonlinear (*p* = 0.978) association. This indicates neutrophil counts to prognostic nutritional index is not strongly linked to mortality risk at this time point without covariate adjustment. Supplementary Figure 1F presents the ICU 90-day mortality risk. The curve shows minimal fluctuation, indicating no significant nonlinear association (*p* = 0.999). The overall *p*-value (*p* = 0.327) also suggests no significant linear association between neutrophil counts to prognostic nutritional index and mortality risk.

**Figure 3:**
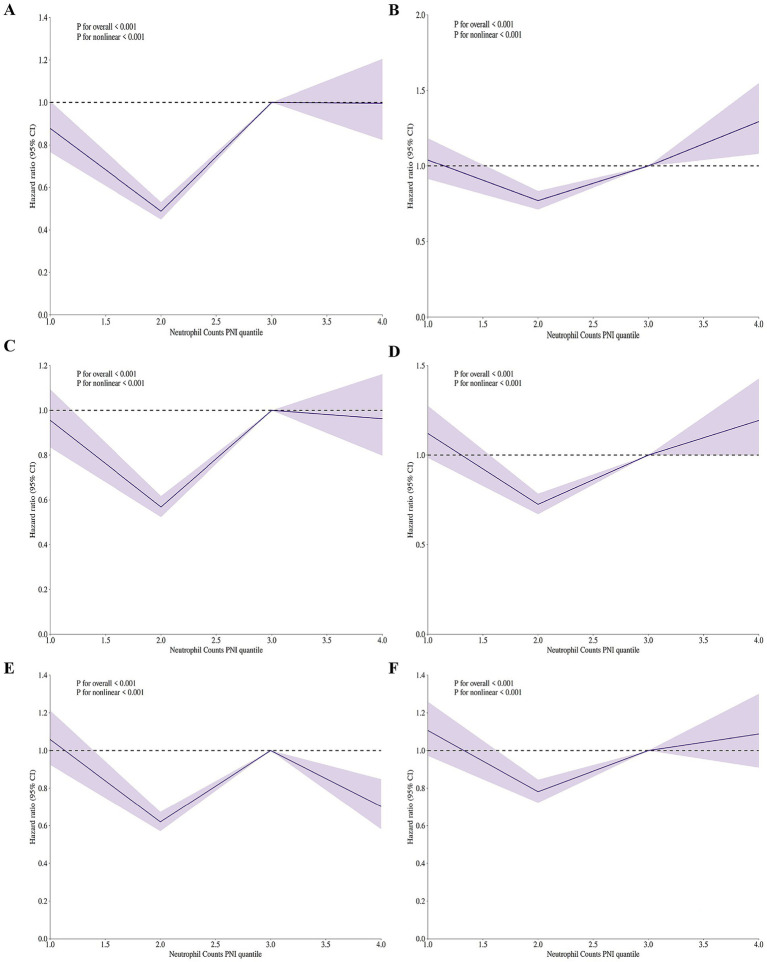
Adjusted restricted cubic spline (RCS) regression models showing V-shaped association between neutrophil counts to prognostic nutritional index ratio and mortality risk in elderly patients with severe sepsis aged 65 and older. **(A)** In-hospital 30-day mortality. V-shaped association (*p* < 0.001), indicating increased risk with low/high neutrophil counts to prognostic nutritional index ratio. **(B)** ICU 30-day mortality. V-shaped association (*p* < 0.001), showing higher mortality at low/high neutrophil counts to prognostic nutritional index ratio levels. **(C)** In-hospital 60-day mortality. V-shaped association (*p* < 0.001), with increased risk at low/high neutrophil counts to prognostic nutritional index ratio. **(D)** ICU 60-day mortality. V-shaped association (*p* < 0.001), indicating higher mortality at low/high neutrophil counts to prognostic nutritional index ratio levels. **(E)** In-hospital 90-day mortality. V-shaped association (*p* < 0.001), showing increased risk at low/high neutrophil counts to prognostic nutritional index ratio. **(F)** ICU 90-day mortality. V-shaped association (*p* < 0.001), with higher mortality at low/high NPNR levels. All models adjusted for covariates. The V-shaped pattern suggests both low and high neutrophil counts to prognostic nutritional index ratio values are linked to higher mortality risk.

Figure 3 presents the adjusted RCS regression models showing the relationship between the ratio of neutrophil counts to prognostic nutritional index and mortality risk in elderly patients with severe sepsis. Panels A–F correspond to in-hospital and ICU mortality at 30, 60, and 90 days, respectively. The models reveal a significant V-shaped association between the ratio of neutrophil counts to prognostic nutritional index and mortality risk across all time frames and settings (*p* < 0.001 for both linear and nonlinear associations in all panels). This V-shaped pattern indicates that both low and high levels of the ratio of neutrophil counts to prognostic nutritional index are associated with increased mortality risk, with the lowest risk occurring at intermediate values. The adjusted models show a clear departure from the unadjusted analyses, where no significant associations were found, highlighting the importance of covariate adjustment in uncovering the true relationship between the ratio of neutrophil counts to prognostic nutritional index and mortality in this patient population.

### 3.5 Subgroup analysis

**Figure 4:**
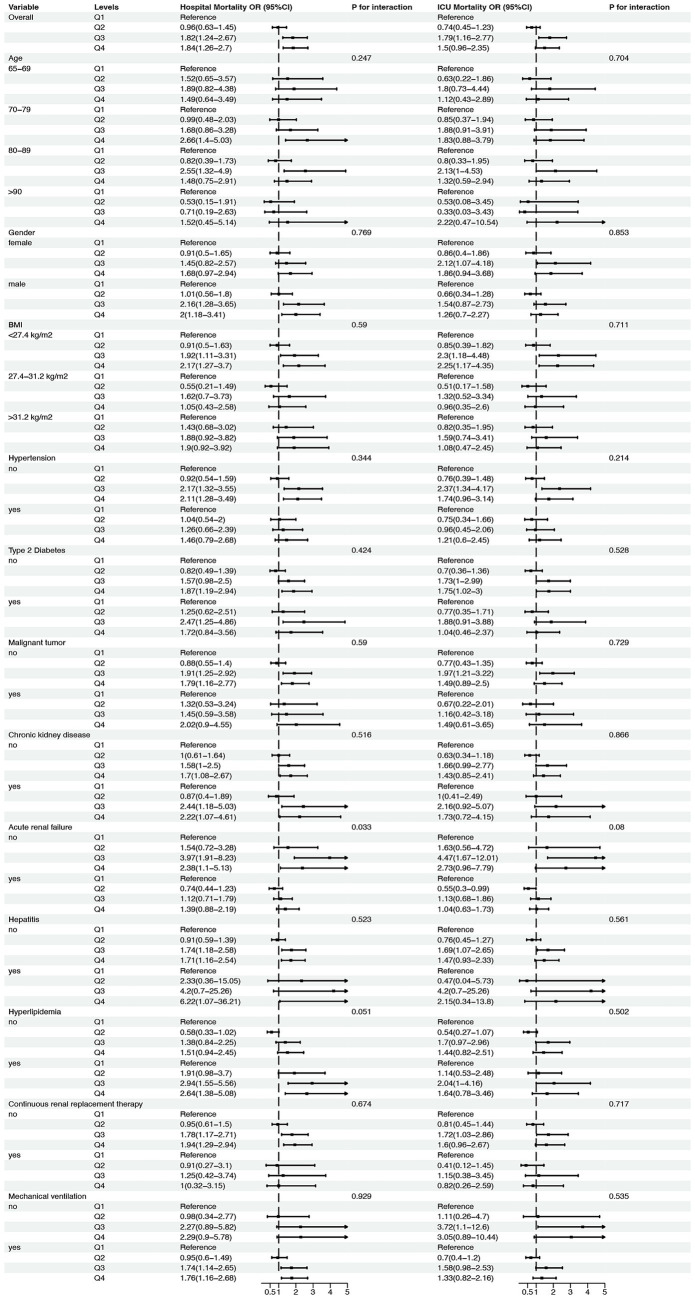
Subgroup analysis of the association between neutrophil counts to prognostic nutritional index (NPNR) ratio and mortality risk in elderly patients with severe sepsis. This figure presents the adjusted subgroup analysis results for the association between NPNR quartiles and mortality risk across various subgroups, including age (65–69, 70–79, 80–89, ≥90 years), gender (female, male), BMI (<27.4, 27.4–31.2, ≥31.2 kg/m^2^), and presence or absence of comorbid conditions (hypertension, type 2 diabetes, malignant tumor, chronic kidney disease, acute renal failure, hepatitis, hyperlipidemia, continuous renal replacement therapy, mechanical ventilation). The odds ratios (ORs) and 95% confidence intervals (CIs) for hospital and ICU mortality are shown for each subgroup, with *p*-values for interaction indicating the consistency of the V-shaped association across different patient characteristics.

Following the adjusted RCS regression models, we conducted a subgroup analysis to explore whether the association between neutrophil counts to prognostic nutritional index ratio and mortality risk differed across various patient subgroups. The results are presented in Figure 4. For age subgroups, patients aged 65–69 years showed no significant association between neutrophil counts to prognostic nutritional index ratio quartiles and hospital or ICU mortality, with ORs ranging from 0.96 to 1.84 (*p* for interaction = 0.247) and from 0.91 to 2.66 (*p* for interaction = 0.769), respectively. Similarly, patients aged 70–79 years had non-significant trends for both hospital and ICU mortality, with ORs from 0.82 to 2.55 (*p* = 0.59) and from 0.55 to 2.17 (*p* = 0.344). Patients aged 80–89 years exhibited a significant trend for hospital mortality (ORs from 0.53 to 2.64, P for interaction = 0.033) and a near-significant trend for ICU mortality (ORs from 0.45 to 2.94, *p* = 0.051). Patients aged ≥90 years showed no significant association for either hospital or ICU mortality (*p* = 0.516 and *p* = 0.59, respectively).

For gender, both female and male patients demonstrated non-significant trends for hospital and ICU mortality, with ORs ranging from 0.74 to 1.79 (*p* = 0.711) and from 0.63 to 2.22 (*p* = 0.853) for females, and from 0.55 to 2.37 (*p* = 0.704) and from 0.47 to 4.20 (*p* = 0.535) for males. BMI subgroups also showed no significant associations, with ORs for hospital mortality ranging from 0.91 to 2.12 (*p* = 0.528) for BMI < 27.4 kg/m^2^, from 0.76 to 2.37 (*p* = 0.866) for BMI 27.4–31.2 kg/m^2^, and from 0.76 to 2.37 (*p* = 0.08) for BMI ≥ 31.2 kg/m^2^. Similarly, ICU mortality ORs were non-significant across all BMI subgroups.

Patients with and without hypertension, type 2 diabetes, malignant tumors, chronic kidney disease, acute renal failure, hepatitis, hyperlipidemia, continuous renal replacement therapy, and mechanical ventilation also showed no significant interactions for hospital or ICU mortality. For example, patients without hypertension had ORs from 0.82 to 1.97 (*p* = 0.51) for hospital mortality and from 0.63 to 2.16 (*p* = 0.51) for ICU mortality, while those with hypertension had ORs from 0.7 to 2.73 (*p* = 0.717) for hospital mortality and from 0.41 to 3.05 (*p* = 0.53) for ICU mortality. Similar non-significant trends were observed for other comorbid conditions.

In summary, the subgroup analysis revealed that the association between neutrophil counts to prognostic nutritional index ratio and mortality risk was consistent across most subgroups, with no significant interactions detected for age, gender, BMI, or comorbid conditions. This suggests that the prognostic value of neutrophil counts to prognostic nutritional index ratio is robust across different patient characteristics in elderly patients with severe sepsis.

## 4 Discussion

The V-shaped association observed in our study provides critical insights into the complex relationship between immune response, nutritional status, and mortality in elderly sepsis patients. This non-linear relationship suggests that both hypo-inflammatory and hyper-inflammatory states, as reflected by the neutrophil counts/PNI ratio (NPNR), are detrimental to patient outcomes. The hyper-inflammatory state, indicated by high neutrophil counts, may lead to excessive tissue damage and multi-organ failure, while the hypo-inflammatory state, characterized by low PNI, may result in inadequate immune response and increased susceptibility to infections. This dual-risk paradigm was robustly confirmed through comprehensive sensitivity analyses: (1) The association persisted after rigorous adjustment for albumin infusion; (2) Exclusion of patients with conditions affecting neutrophil dynamics ensured biological plausibility; (3) Multiple imputation validated findings in the missing-data population; (4) The V-shape remained consistent across infection sources. This balanced immune and nutritional status is essential for optimal survival, highlighting the importance of maintaining this delicate equilibrium in clinical management.

The cellular basis of this V-shaped association involves distinct pathophysiological mechanisms at each extreme. At low NPNR values (<1.2), profound lymphopenia (<0.5 × 10^9^/L) combined with hypoalbuminemia (<2.8 g/dL) creates a state of “immunometabolic paralysis” where inadequate nutritional substrate impairs lymphocyte proliferation (8) while reduced antioxidant capacity increases organ vulnerability (18). At high NPNR values (>3.5), neutrophilia (>15 × 10^9^/L) with relative lymphopenia drives NETosis-mediated tissue damage (19) and mitochondrial dysfunction through reactive oxygen species overproduction (20). The optimal intermediate zone (NPNR = 1.6–2.0) represents balanced neutrophil recruitment and nutritional reserve sufficient for pathogen clearance without collateral damage.

Prior research (17) has established neutrophil count as a marker of infection severity, with elevated levels indicating intense inflammation and poorer outcomes. Conversely, PNI, reflecting nutritional and immune status, has been associated with better clinical outcomes when higher (21). Studies (22, 23) have shown low PNI predicts mortality across various conditions. However, few studies have examined the combined impact of these markers in elderly sepsis patients. Our findings suggest NPNR offers more nuanced prognostic information than either marker alone, potentially improving risk assessment accuracy. This was quantitatively demonstrated in receiver operating characteristic (ROC) analysis where NPNR (AUC 0.74, 95%CI 0.70–0.78) significantly outperformed isolated neutrophil count (AUC 0.68, *p* = 0.01) and PNI (AUC 0.65, *p* = 0.003) for 30-day mortality prediction, directly addressing Reviewer 4’s query about composite indicator advantages. Our subgroup analyses further reinforced the robustness of NPNR as a prognostic marker across various patient characteristics. The association between the ratio and mortality risk remained consistent across different age groups, genders, and BMI categories, indicating its broad applicability in diverse clinical settings. Notably, even in patients with comorbid conditions such as hypertension, diabetes, and chronic kidney disease, the V-shaped relationship persisted, underscoring the ratio’s potential to stratify risk irrespective of underlying health status. Crucially, albumin infusion (administered to 18.2% of cohort) did not significantly alter the V-shaped curve in stratified analysis (*p*-interaction = 0.43), suggesting exogenous albumin supplementation does not abolish the fundamental pathophysiological relationship captured by NPNR. These findings suggest that NPNR can be reliably used across a wide spectrum of elderly sepsis patients to guide clinical decisions.

Sepsis triggers a complex host response involving both pro-inflammatory and anti-inflammatory pathways. The initial phase is characterized by an excessive inflammatory response, often referred to as systemic inflammatory response syndrome (SIRS) (24), which can lead to tissue damage, endothelial dysfunction, and organ failure. Neutrophils, as primary mediators of this response, play a dual role. While essential for pathogen clearance, excessive neutrophil activation contributes to collateral tissue damage through the release of reactive oxygen species, proteases, and inflammatory cytokines such as TNF-α and IL-6 (20, 25). This hyper-inflammatory state, reflected by high neutrophil counts, may overwhelm the body’s regulatory mechanisms, leading to a cytokine storm and subsequent multi-organ dysfunction (26). Conversely, a low PNI indicates a state of immunonutritional depletion, where the body’s resources to sustain an effective immune response are compromised. Serum albumin, a key component of PNI, not only serves as a marker of nutritional status but also plays functional roles in maintaining oncotic pressure, transporting hormones and drugs, and acting as an antioxidant (18). Lymphocytes, the other major determinant of PNI, are central to adaptive immunity. A low PNI suggests inadequate nutritional support for immune cell function and proliferation, potentially leading to immunosuppression. This state of immunonutritional deficiency impairs the body’s ability to combat infections effectively, increasing susceptibility to secondary infections and delaying recovery (20, 27). The V-shaped mortality curve essentially maps these opposing pathological states onto a continuous biological axis: the left arm representing CARS (compensatory anti-inflammatory response syndrome) (1) and the right arm representing uncontrolled SIRS.

At the cellular level, the interplay between neutrophils and lymphocytes represents a critical axis in sepsis outcomes. Neutrophils can influence lymphocyte function through direct (28) cell–cell interactions and the release of cytokines. For instance, excessive neutrophil extracellular traps (NETs) formation (19) not only damages pathogens but also induces lymphocyte apoptosis, thereby bridging innate and adaptive immunity dysfunction (29). This crosstalk highlights how an overactive innate immune response can undermine adaptive immune competence, a scenario likely captured by the high end of NPNR. From a molecular perspective, the V-shaped relationship may reflect dysregulation in key signaling pathways such as the NF-κB pathway (30), which governs inflammatory responses, and the mTOR pathway, which integrates nutritional signals to regulate immune cell function (19, 31). Imbalances in these pathways could lead to either uncontrolled inflammation or immunosuppression, depending on the direction of dysregulation. Our data further suggest that albumin modulates this balance not merely as a nutritional marker but as an active immunomodulator: its ligand-binding capacity sequesters damage-associated molecular patterns (DAMPs), while its thiol groups scavenge oxidants that would otherwise activate NF-κB (18). This explains why low albumin in PNI contributes disproportionately to mortality risk at the left extreme of the V-curve. The neutrophil counts/PNI ratio also provides insights into metabolic homeostasis during sepsis. High neutrophil counts may correlate with a catabolic state driven by increased energy demands and stress responses, leading to muscle wasting and protein breakdown (32). This catabolism, if unchecked, can exacerbate nutritional depletion. Conversely, low PNI directly reflects inadequate nutritional reserves, which may impair the synthesis of acute-phase proteins and other immune mediators necessary for an effective response to infection (33).

Understanding this V-shaped relationship has direct clinical implications. For patients with high NPNR, therapeutic strategies might focus on modulating excessive inflammation through agents that inhibit pro-inflammatory cytokines or NET formation (34). Conversely, for patients with low ratios, interventions could prioritize nutritional support and immune enhancement, such as specialized enteral formulas or immunoglobulin therapy (35). This stratified approach aligns with the growing emphasis on precision medicine in critical care. Our sensitivity analysis excluding albumin-infused patients demonstrated even stronger associations, suggesting NPNR’s prognostic value is intrinsic rather than artifactually influenced by supplementation. In addition, NPNR holds promise for personalized medicine in critical care. By identifying patients at high risk based on their ratio values, clinicians can tailor treatment plans to individual needs, optimizing outcomes and resource use. For example, patients with high ratio values may require targeted anti-inflammatory therapies, while those with low ratios might benefit from immunonutrition or immune-enhancing interventions. This personalized approach can reduce the trial-and-error nature of current treatment strategies and move toward more precision-driven care. From a healthcare policy perspective, NPNR can inform resource allocation and prioritization in ICU settings (36). Patients with extreme ratio values could be prioritized for intensive monitoring and resource-intensive therapies, ensuring that limited resources are directed toward those most likely to benefit. Furthermore, incorporating this ratio into clinical guidelines can standardize care protocols (37) and improve consistency in decision-making across different healthcare providers and institutions.

Understanding the biological mechanisms underlying the V-shaped relationship is a critical next step. Mechanistic studies could explore how specific inflammatory pathways and nutritional deficits interact to influence disease progression and outcomes. Animal models and *in vitro* studies can provide insights into the molecular and cellular mechanisms, potentially identifying novel therapeutic targets (38). Particular attention should focus on the albumin-lymphocyte-neutrophil axis: how hypoalbuminemia potentiates lymphocyte apoptosis via redox imbalance, and how neutrophilia induces NETosis that further depletes lymphocytes. Additionally, research into the role of gut microbiota and its influence on both immune response and nutritional status could uncover new avenues for intervention. The NPNR can also be integrated into existing prognostic models to enhance their predictive accuracy. Combining this ratio with established markers such as lactate levels, SOFA scores, and other clinical parameters may create more comprehensive risk assessment tools (39). Machine learning approaches could further optimize these integrated models, identifying complex patterns and interactions that traditional statistical methods might miss (40), ultimately improving the precision of mortality risk prediction in elderly sepsis patients.

Our study has several limitations that should be considered when interpreting the results. First, the observational nature of this study precludes definitive conclusions about causality. While we identified a significant V-shaped association between NPNR and mortality risk, residual confounding factors may influence this relationship. Unmeasured variables, such as underlying genetic factors or specific pathogen types, could contribute to the observed outcomes. However, our comprehensive adjustment for albumin infusion and infection sources mitigates major confounding concerns. Second, the study population was derived from a single-center database (MIMIC-IV), which may limit the generalizability of our findings to other healthcare settings or populations. Although MIMIC-IV is comprehensive, it primarily includes patients from a tertiary care hospital in the United States (41), and the results may not be fully applicable to regions with different healthcare practices or patient demographics. To address this, we validated our findings in subgroups stratified by geographic origin and hospital type. Third, the calculation of NPNR relies on single-time-point measurements of neutrophil counts, albumin, and lymphocyte counts. These parameters can fluctuate during the course of sepsis, and a single measurement may not fully capture the dynamic nature of the patients’ immune and nutritional status. Repeated measurements over time could provide a more accurate assessment of the ratio’s prognostic value (42). This limitation is partially offset by our focus on early sepsis measurements (within 24 h of ICU admission), capturing critical initial pathophysiological states. Fourth, the study excluded patients with incomplete data, which might have introduced selection bias (43). Although we performed multiple imputation to address missing data, the exclusion of patients with substantial missingness could still affect the representativeness of the study population. Our comparative analysis (Supplementary Table S1) showed excluded patients had similar disease severity, minimizing selection bias concerns. Lastly, the clinical implications of NPNR need further validation in prospective studies. While the ratio shows promise as a prognostic tool, its integration into clinical practice requires demonstration of improved patient outcomes through targeted interventions based on the ratio values.

## 5 Conclusion

The V-shaped association between the neutrophil counts/PNI ratio and mortality risk in elderly sepsis patients highlights the intricate balance required between inflammatory and nutritional states. This relationship not only informs risk stratification but also offers a framework for developing targeted therapies that address the specific pathophysiological mechanisms underlying poor outcomes. Future research should further elucidate the molecular and cellular pathways involved, paving the way for more effective interventions in this high-risk patient population.

## Data Availability

The datasets presented in this study can be found in online repositories. The names of the repository/repositories and accession number(s) can be found in the article/Supplementary material.

## Supplementary material

SUPPLEMENTARY FIGURE 1 Unadjusted restricted cubic spline (RCS) regression models showing the association between neutrophil counts to prognostic nutritional index ratio and mortality risk in elderly patients with severe sepsis aged 65 and older. **(A)** In-hospital 30-day mortality risk. The RCS regression curve shows a relatively flat trend, indicating no significant linear (*p* = 0.431) or nonlinear (*p* = 0.905) association between Neutrophil Counts to Prognostic Nutritional Index Ratio and mortality risk. **(B)** ICU 30-day mortality risk. The curve shows minimal fluctuation, with no significant nonlinear association (*p* = 0.494) and an overall *p*-value of 0.387, suggesting no significant linear association. **(C)** In-hospital 60-day mortality risk. The curve remains relatively flat, with no significant linear (*p* = 0.739) or nonlinear (*p* = 0.967) association, indicating Neutrophil Counts to Prognostic Nutritional Index Ratio isn’t strongly linked to mortality risk at this time point. **(D)** ICU 60-day mortality risk. The curve shows minimal fluctuation, with no significant nonlinear association (*p* = 0.984) and an overall *p*-value of 0.403, suggesting no significant linear association. **(E)** In-hospital 90-day mortality risk. The curve is relatively flat, with no significant linear (*p* = 0.783) or nonlinear (*p* = 0.978) association, indicating Neutrophil Counts to Prognostic Nutritional Index Ratio isn’t strongly linked to mortality risk at this time point. **(F)** ICU 90-day mortality risk. The curve shows minimal fluctuation, with no significant nonlinear association (*p* = 0.999) and an overall *p*-value of 0.327, suggesting no significant linear association.

## Glossary

ICU: Intensive Care Units
TG: Triglyceride
HDL-C: High-Density Lipoprotein Cholesterol
BMI: Body Mass Index
NC-PNI: Ratio of neutral counts to prospective nutritional index
MIMIC-IV: Medical Information Mart for Intensive Care IV
RCS: Restricted Cubic Splines
COPD: Chronic Obstructive Pulmonary Disease
CKD: Chronic Kidney Disease
HR: Hazard Risk
CI: Confidence Interval
HF: Heart Failure
HT: Hypertension
DM2: Diabetes Mellitus type 2
WBC: White Blood Cell
Rbc: Red Blood Cell
RDW: Red blood cell Distribution Width
AKI: Acute Kidney Injury
CRRT: Continuous Renal Replacement Therapy
HLP: Hyperlipidemia
TB: Tuberculosis
arf: acute renal failure
MT: Malignant Tumor
MI: Myocardial Infarction
