# Supplementary figures and images for: The V-shaped association between the ratio of neutrophil counts to prognostic nutritional index and 30-, 60-, and 90-day mortality in elderly critically ill patients aged 65 and older with sepsis: a retrospective study based on the MIMIC database
Source: Front Nutr. 2025 Sep 3;12:1602016. doi: 10.3389/fnut.2025.1602016 (PMC12442426; doi:10.3389/fnut.2025.1602016)

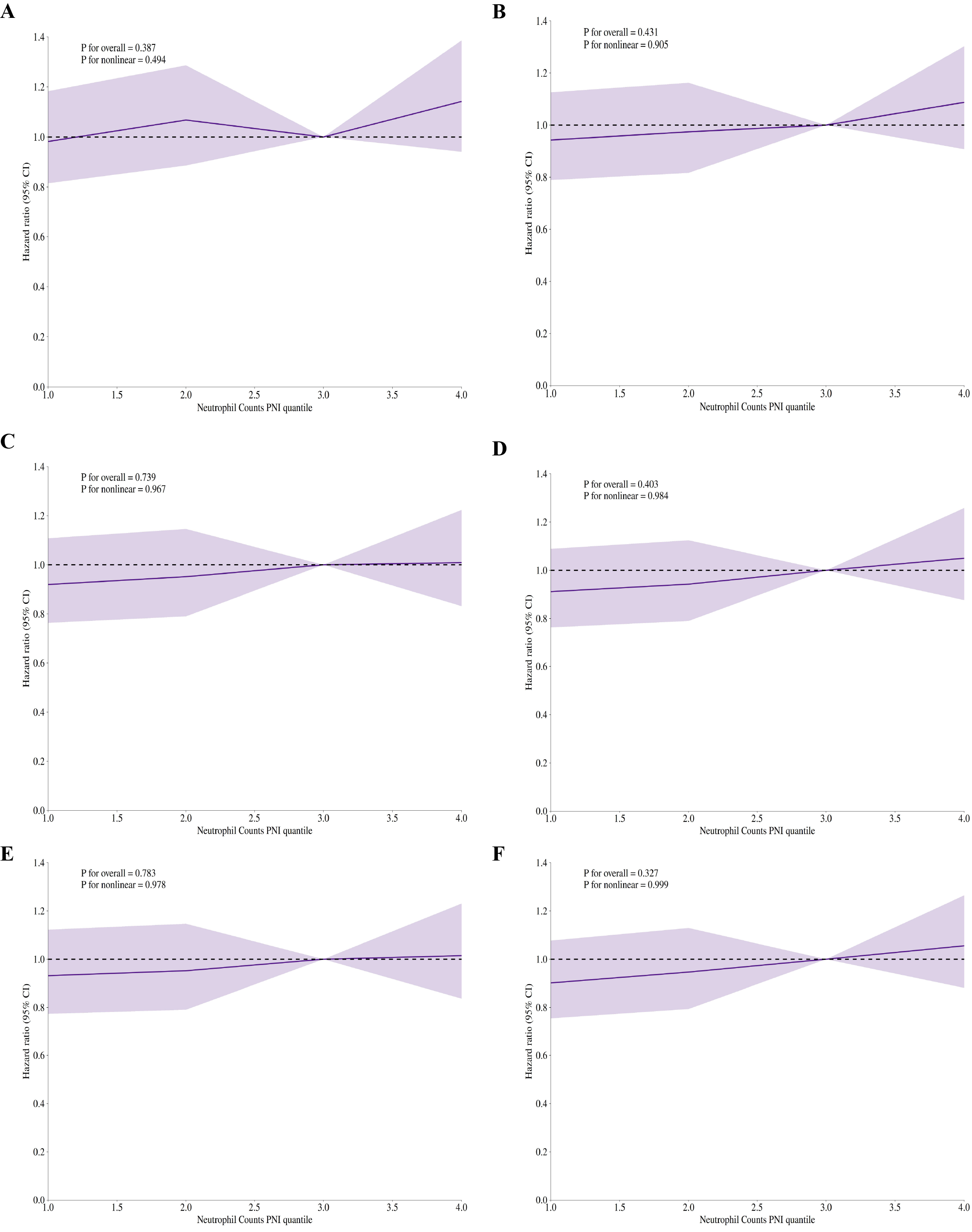

Supplement: SUPPLEMENTARY FIGURE 1 — Unadjusted restricted cubic spline (RCS) regression models showing the association between neutrophil counts to prognostic nutritional index ratio and mortality risk in elderly patients with severe sepsis aged 65 and older. (A) In-hospital 30-day mortality risk. The RCS regression curve shows a relatively flat trend, indicating no significant linear (p = 0.431) or nonlinear (p = 0.905) association between Neutrophil Counts to Prognostic Nutritional Index Ratio and mortality risk. (B) ICU 30-day mortality risk. The curve shows minimal fluctuation, with no significant nonlinear association (p = 0.494) and an overall p-value of 0.387, suggesting no significant linear association. (C) In-hospital 60-day mortality risk. The curve remains relatively flat, with no significant linear (p = 0.739) or nonlinear (p = 0.967) association, indicating Neutrophil Counts to Prognostic Nutritional Index Ratio isn’t strongly linked to mortality risk at this time point. (D) ICU 60-day mortality risk. The curve shows minimal fluctuation, with no significant nonlinear association (p = 0.984) and an overall p-value of 0.403, suggesting no significant linear association. (E) In-hospital 90-day mortality risk. The curve is relatively flat, with no significant linear (p = 0.783) or nonlinear (p = 0.978) association, indicating Neutrophil Counts to Prognostic Nutritional Index Ratio isn’t strongly linked to mortality risk at this time point. (F) ICU 90-day mortality risk. The curve shows minimal fluctuation, with no significant nonlinear association (p = 0.999) and an overall p-value of 0.327, suggesting no significant linear association. [file Image_1.TIF]
