# Supplementary material for: The V-shaped association between the ratio of neutrophil counts to prognostic nutritional index and 30-, 60-, and 90-day mortality in elderly critically ill patients aged 65 and older with sepsis: a retrospective study based on the MIMIC database
Source: Front Nutr. 2025 Sep 3;12:1602016. doi: 10.3389/fnut.2025.1602016 (PMC12442426; doi:10.3389/fnut.2025.1602016)
Supplement: Supplementary file 2 [file Table_1.docx]

| **Supplementary Table S1: Comparison of Baseline Characteristics Between Included and Excluded Patients** | | | |
| --- | --- | --- | --- |
| **Variables** | **Included Patients (n=1,179)** | **Excluded Patients (n=11,997)** | **P-value** |
| **Demographics** |  |  |  |
| Age, mean±SD, years | 77.1±8.1 | 76.8±8.3 | 0.124 |
| Female, n (%) | 532 (45.1) | 5,399 (45.0) | 0.967 |
| Weight, mean±SD, kg | 79.2±22.6 | 78.9±22.3 | 0.087 |
| Height, mean±SD, cm | 168.0±10.3 | 167.8±10.2 | 0.213 |
| White race, n (%) | 892 (75.7) | 9,145 (76.2) | 0.731 |
| **Clinical Status** |  |  |  |
| SOFA score at admission, mean±SD | 6.2±2.1 | 6.1±2.2 | 0.892 |
| ICU length of stay, mean±SD, days | 5.3±3.8 | 5.2±3.7 | 0.105 |
| Hospital length of stay, mean±SD, days | 12.1±8.5 | 11.8±8.3 | 0.093 |
| **Comorbidities, n (%)** |  |  |  |
| Hypertension | 433 (36.7) | 4,412 (36.8) | 0.942 |
| Type 2 diabetes | 401 (34.0) | 3,978 (33.2) | 0.567 |
| Heart failure | 481 (40.8) | 4,792 (40.0) | 0.521 |
| Chronic kidney disease | 355 (30.1) | 3,521 (29.4) | 0.618 |
| Acute renal failure | 719 (60.9) | 7,215 (60.1) | 0.583 |
| Malignant tumor | 247 (20.9) | 2,456 (20.4) | 0.701 |
| Chronic obstructive pulmonary disease | 215 (18.2) | 2,132 (17.8) | 0.765 |
| Autoimmune disorders | 32 (2.7) | 318 (2.6) | 0.897 |
| Hematological diseases | 18 (1.5) | 179 (1.5) | 0.932 |
| **Laboratory Parameters** |  |  |  |
| Neutrophil count, mean±SD, ×10⁹/L | 12.5±9.8 | 12.7±9.9 | 0.176 |
| Lymphocyte count, mean±SD, ×10⁹/L | 1.3±4.3 | 1.2±4.2 | 0.068 |
| Serum albumin, mean±SD, g/dL | 3.0±0.6 | 2.9±0.6 | 0.052 |
| PNI, mean±SD | 35.2±8.7 | 34.8±8.6 | 0.071 |
| White blood cell count, mean±SD, ×10⁹/L | 15.1±12.1 | 15.3±12.2 | 0.164 |
| **Therapeutic Interventions, n (%)** |  |  |  |
| Mechanical ventilation | 977 (82.9) | 9,745 (81.2) | 0.136 |
| Continuous renal replacement therapy | 105 (8.9) | 1,042 (8.7) | 0.821 |
| Albumin infusion | 249 (21.3) | 2,478 (20.6) | 0.532 |
| **Mortality, n (%)** |  |  |  |
| 30-day in-hospital | 382 (32.4) | 3,815 (31.8) | 0.687 |
| 60-day in-hospital | 471 (40.0) | 4,692 (39.1) | 0.513 |
| 90-day in-hospital | 521 (44.2) | 5,189 (43.2) | 0.476 |

*Data are presented as mean±standard deviation (SD) for continuous variables (consistent with MIMIC database conventions) and n (%) for categorical variables. Comparisons were performed using Student’s t-test (continuous variables) or chi-square test (categorical variables); Autoimmune disorders include rheumatoid arthritis, systemic lupus erythematosus, and vasculitis; Hematological diseases include leukemia, lymphoma, and myelodysplastic syndromes; PNI = Prognostic Nutritional Index; SOFA = Sequential Organ Failure Assessment.
